# Artificial Intelligence and Modern Technology in Dentistry: Attitudes, Knowledge, Use, and Barriers Among Dentists in Croatia—A Survey-Based Study

**DOI:** 10.3390/clinpract14060207

**Published:** 2024-12-05

**Authors:** Ana Ivanišević, Antonija Tadin

**Affiliations:** 1 Department of Restorative Dental Medicine and Endodontics, Study of Dental Medicine, School of Medicine, University of Split, 21000 Split, Croatia; ivanisevicana266@gmail.com; 2 Department of Maxillofacial Surgery, Clinical Hospital Centre Split, 21000 Split, Croatia

**Keywords:** dental medicine, artificial intelligence, modern technology, knowledge assessment, clinical practice, barriers to implementation

## Abstract

Aim: This study aims to assess Croatian dentists’ knowledge, attitudes, and use of artificial intelligence (AI) and modern technology, while also identifying perceived barriers to AI and modern technology adoption and evaluating the need for further education and training. Materials and Methods: A cross-sectional survey was conducted in February 2024 among general dentists in Croatia using a self-structured questionnaire. A total of 200 respondents filled out the questionnaire. It included five sections: socio-demographic and professional information, self-assessment of AI and modern technology use, knowledge of AI in dentistry, current innovations and devices used in practice, and barriers to AI and modern technology integration in practice. Data were analyzed using descriptive statistics and a regression analysis to explore relationships between socio-demographic factors and AI knowledge. Results: The mean knowledge of AI systems was 3.62 ± 2.56 out of a possible score of 7, indicating relatively poor knowledge, with 47.5% demonstrating knowledge below the median. Most respondents (76.0%) did not use AI systems and modern technology in practice; however, prosthodontics (13.0%) and oral surgery (10.0%) were identified as the primary fields utilizing these technologies. Respondents rated their knowledge of modern technologies and AI as weak or moderate, with 60.5% engaged in continuous education. Despite 76.0% not using AI daily, 71.0% believed that these technologies could enhance patient care. Participants interested in further training showed significantly better knowledge of AI applications (*p* = 0.030). Major barriers included acquisition and maintenance costs (59.0%) and financial constraints (58.0%). Conclusions: The study revealed that most respondents had poor knowledge of AI systems. Despite this, there is a recognition of AI’s and modern technology potential in dentistry, emphasizing the need for enhanced education and training in this field.

## 1. Introduction

Artificial intelligence (AI) and related technologies are increasingly present in society and the economy and are beginning to be used in healthcare. These technologies enable the transformation of healthcare management processes between doctors and pharmaceutical companies. Numerous scientific studies already indicate that artificial intelligence may perform some healthcare tasks as well or better than humans, for example, in the diagnosis of diseases [1,2]. There is no question as to whether these modern technologies will be sufficiently “capable” to be useful. Rather, the challenge for AI is whether it will be adopted in clinical practice and how long it will take before it is used on a larger scale. To accelerate their adoption, AI systems must be integrated into electronic health records, quality training must be provided within public or private organizations, and information about them must be regularly updated. It is assumed that artificial intelligence systems will not significantly replace humans in medical processes, or if they do, it will take a long time because artificial intelligence does not possess unique human skills such as empathy, persuasion, and big-picture integration. Nevertheless, artificial intelligence can help doctors to invest more effort in the treatment of their patients [1].

The advantages of using artificial intelligence systems include faster work and higher profits, ease of use, the ability to work without a break, solving complicated and complex problems, reducing human error (if the system is programmed correctly), and unlimited functionality. Moreover, since emotional factors are excluded in machines, a system with artificial intelligence can deliver results and make decisions much faster. In medicine, it has a growing potential to assist in classification, screening of patients, diagnoses, decision making, prognoses, and long-term monitoring and can be used to assist in drug development and clinical analyses. The disadvantages of AI systems include expensive equipment and repairs, rising unemployment, the automation of jobs, inability to create a human connection and team, increasing reliance on technology among younger generations, and a lack of personal touch. An additional limiting factor is access to data, as some facilities do not share their data and in some regions, there is a lack of medical information that makes it difficult to combine data between facilities. Making wrong or incomplete decisions that occur due to incorrect data entry is one of the standard flaws of artificial intelligence and this could be a big problem in medicine and treating patients [2,3,4].

Artificial intelligence is used in dentistry for numerous purposes. Some of the areas in which it is used are dental radiology (analysis of X-rays and CT scans), treatment planning, prosthodontics (fabrication of dentures), periodontics (diagnosis of periodontitis), endodontics (detection of canal morphology, lesions, or fractures), orthodontics (treatment planning), forensic dentistry, oral pathology (detection of tumor tissue), dental robotics, and others [5]. Worldwide, there are numerous studies on the application of artificial intelligence in dentistry, in which the attitude, knowledge, and application of artificial intelligence among students [6,7,8,9], dentists [6,7,8,9,10,11,12], and dental assistants [13] were investigated. In most studies, respondents emphasize that their knowledge of artificial intelligence is insufficient. The respondents mostly have a positive and optimistic attitude towards artificial intelligence and believe that more education is needed to increase the use of artificial intelligence in clinical practice. They also conclude that the integration of artificial intelligence into the dental curriculum, i.e., dental education, is necessary. Such research, presentations, and publications can significantly contribute to raising awareness of artificial intelligence [6,7,8,9,11]. While artificial intelligence remains in the nascent phases of its evolution, its capacity to enhance diagnostic accuracy, optimize treatment strategies, and ultimately improve patient outcomes is irrefutable. As AI technologies progress, it becomes increasingly essential for dentists and dental students to remain informed about the latest innovations and cultivate a robust understanding of digital technologies [14]. According to Tomášik et al., with careful integration, continued human oversight and input, and ethical management, AI technologies can revolutionize and greatly support orthodontic treatment planning, leading to more personalized and effective patient care [15].

The Croatian government has been developing a national strategy for artificial intelligence since 2021, with a working group of experts tasked to draft the National Plan for its development. Members of the Croatian Association for Artificial Intelligence (CroAI) emphasize the need for Croatia to actively engage in AI integration into education to avoid falling behind, similar to the missed opportunities with the Internet. They advocate for a shift in mindset to overcome the fear of the unknown and stress the importance of effectively teaching students to utilize AI systems, ensuring sufficient expertise in this field [16,17].

The purpose of this research was to examine the knowledge, attitudes, and practices related to modern technology and artificial intelligence (AI) among dental practitioners in Croatia. Given the increasing integration of these technologies in healthcare, this study addressed a notable gap in the literature, as no prior research had focused on this topic within the Croatian dental community. Specifically, the study aimed to assess dental practitioners’ understanding of AI, their attitudes toward its application, and the barriers they encountered in implementing these technologies. The hypothesis posited that dental practitioners in Croatia possessed insufficient knowledge of AI and modern technologies, and that their practical application of these technologies was also inadequate.

## 2. Materials and Methods

### 2.1. Study Design and Study Population

This cross-sectional study was conducted in February 2024 at the Department of Restorative Dental Medicine and Endodontics, School of Dental Medicine, with the approval of the Ethics Committee of the School of Medicine, University of Split (Class: 003-08/23-03/0015, Ref. No.: 2181-198-03-04-23-0079). The study was conducted in accordance with the institutional code of ethics and the Declaration of Helsinki.

Data were collected using a questionnaire distributed to dentists in digital or paper form during the “Dentistry Today” congress held in Split on 16–17 February 2024. At the beginning of the questionnaire, respondents were informed that participation in the survey was anonymous and voluntary. Respondents could withdraw from the survey at any time without giving a reason. The completion of the questionnaire was regarded as consent to participate in the study. No data were collected in the survey that would reveal the identity of the respondents, only information on education, years of work experience, gender, and workplace.

The minimum necessary sample size (N = 152) was calculated using the Sample Size Calculator (Inc. RaoSoft^®^, Seattle, WA, USA), based on an estimated population of dentists attending the conference (N = 250), an expected response rate of 50%, a confidence level of 95%, and a margin of error of 5%. A convenience sampling method was utilized for this study.

The inclusion criteria for this study required participants to be dentists practicing in the Republic of Croatia with a minimum of one year of clinical experience. Additionally, participants had to be willing to participate and fully complete the questionnaire. Conversely, the exclusion criteria encompassed unemployed dentists, those without clinical experience, dentists practicing outside the Republic of Croatia, and specialists from various dental fields.

### 2.2. Questionnaire

The questionnaire was designed based on a review of the relevant literature on this topic [7,8,9,10,18] and consisted of five main sections with a total of 44 questions. The questionnaire was developed by a dental student and a professor of restorative dentistry and endodontics. After a thorough review and pilot testing with ten dentists, adjustments were made based on feedback regarding its clarity. The questionnaire took 10 to 15 min to complete.

The first section consisted of five questions about the respondent’s socio-demographic and professional information, including gender, age, workplace, years of clinical experience, and education level (Q1–Q5).

The second section contained seven questions on respondents’ self-assessment of their use of artificial intelligence and advanced technology in their clinical practice (Q6–Q12). Respondents were asked to indicate the extent to which they currently use artificial intelligence and modern technologies, the extent to which they plan to use them in the future, and whether they see the potential to improve their work through the application of these technologies and additional training. The answers to four questions were “Yes”, “No”, and “I don’t know”, while three questions offered a response scale from “Not at all” to “Excellent”. Questions were also asked about the areas of dentistry where respondents most frequently use these technologies (Q13–Q24). Twelve areas of dentistry were listed and respondents ticked “Yes” or “No” depending on their use.

The third section consisted of seven questions designed to determine how much dentists currently know about modern technology and artificial intelligence in the field of dental medicine (Q25–Q31). The questions were specific (all of them are listed in tables in the Results section) and respondents indicated whether they believed that modern technology and artificial intelligence were being used in a particular branch of dentistry and for a particular purpose (“Yes”, “No”, and “Don’t know”). The average knowledge of respondents was determined by the sum of their “Yes” responses, leading to a total knowledge score. Based on Bloom’s taxonomy, participants scoring 80–100% were categorized as having “good knowledge”, those scoring 60–79% had “moderate knowledge”, and scores below 60% indicated “poor knowledge”.

The fourth section contained questions about what innovations and devices dentists currently use in their clinical practice (Q32–Q34). This section had three subsections: information on the use of these technologies in diagnostics and treatment planning, therapy, and other innovations. A total of forty-six innovations were listed, and respondents answered “Yes” or “No” depending on their use.

The final section listed ten barriers and disadvantages that respondents encounter or could potentially encounter when trying to integrate artificial intelligence and modern technologies into their clinical practice (Q35–Q44). Respondents could give their answers on a five-point Likert scale, ranging from “strongly disagree” to “strongly agree” with the stated obstacle.

### 2.3. Data Analysis

Statistical data processing was conducted using IBM SPSS Statistics, version 26.0 (SPSS, IBM Corp, Armonk, NY, USA). The Kolmogorov–Smirnov test was applied to evaluate the normality of the distribution of responses. A descriptive analysis presented variables as percentages and numbers, along with the odds ratio (OR) and a 95% confidence interval (CI). The significance of the results was determined at a *p*-value of less than 0.05. Additionally, linear general regression was employed to examine the relationship between respondents’ socio-demographic and occupational data and their knowledge level regarding modern technologies and artificial intelligence.

## 3. Results

Table 1 presents the socio-demographic and occupational data of the respondents. A total of 200 respondents participated in the study, with 74.5% (N = 149) being female. All respondents were general dentists, among whom 4.0% (N = 8) held a doctoral degree and 6.5% (N = 13) held a master’s degree. The majority of participants were between 23 and 40 years old (N = 120, 60.0%). Sixty percent of the respondents (N = 120) had up to 10 years of experience in clinical dentistry. Of the respondents, 47.0% worked in a health center (N = 94), 39.0% (N = 78) were in private practice, and 14.0% (N = 28) worked in a concession. According to the socio-demographic characteristics, women exhibited a lower level of knowledge about the use of AI in dentistry compared to men (*p* ≤ 0.001), while dentists with a doctorate demonstrated greater knowledge than those with only a dental degree (*p* = 0.024).

Table 2 presents data on the self-assessment of knowledge, understanding, and use of modern technologies and artificial intelligence in clinical practice. The majority of respondents rated their current knowledge and understanding of these technologies as weak (N = 56, 28.0%) or moderate (N = 73, 36.0%). Notably, 60.5% (N = 120) of respondents engaged in continuous training and education on these topics, and 78.5% (N = 157) expressed willingness to participate in additional training to enhance their understanding and use of modern technologies and artificial intelligence in the future. Most respondents believed that the implementation of these technologies could improve the quality of patient care (N = 142, 71.0%). Furthermore, participants interested in additional education demonstrated better knowledge of these topics compared to those who were not (*p* = 0.030).

**Figure 1:**
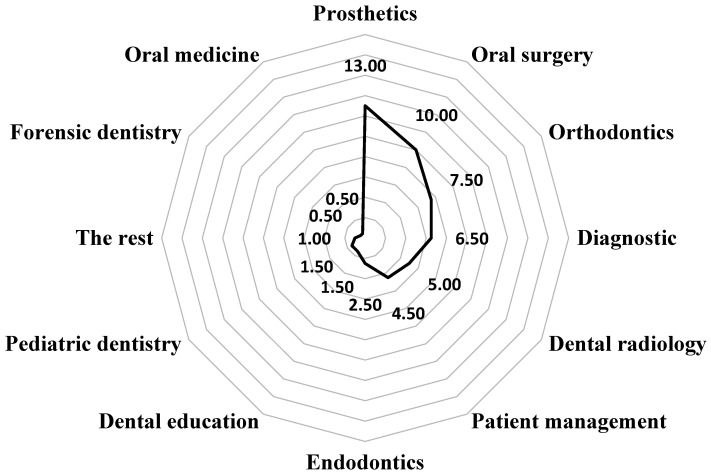
“In which branches of dental medicine have you used AI systems?” (% of responses).

Figure 1 shows data on the use of modern technologies and AI systems in the clinical practice of respondents in specific areas of dentistry. It indicates that 76.0% (N = 152) of respondents do not utilize these technologies in their daily clinical practice, while their main applications are found in prosthodontics (13.0%, N = 26) and oral surgery (10.0%, N = 20).

**Figure 2:**
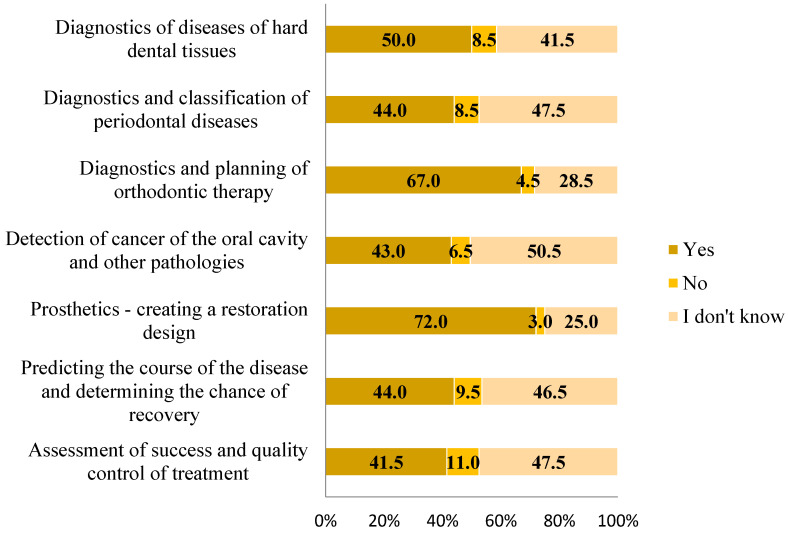
Percentages of responses to the questions whether respondents know that AI can be used for the purposes in dental medicine indicated in the figure.

Figure 2 shows the participants’ responses regarding their knowledge of the use of modern technologies and artificial intelligence in specific branches and areas of dentistry. The majority of participants believe that these technologies can be applied in dental prosthetics (N = 144, 72.0%) for creating restoration designs and in orthodontics (N = 134, 67.0%) for treatment planning and predicting outcomes, such as simulating changes in facial photos before and after treatment. The mean knowledge of AI systems was 3.62 ± 2.56 (Md = 4.00, ICR = 1.25–6.00, min = 0.00, max = 7.00), with 47.5% (N = 95) having knowledge below the median.

Table 3 shows data on the use of individual innovations in modern technology and artificial intelligence for diagnostics and treatment planning. Among all innovations, digital imaging (N = 142, 71.0%) and cone beam computed tomography (CBCT) systems (N = 134, 67.0%) were cited as the most commonly used tools in the respondents’ clinical practice for diagnostic purposes.

Table 4 shows data on the use of specific innovations in patient therapy. Among the innovations most frequently utilized in therapy, intraoral scanners for fixed prosthodontics (N = 63, 31.5%), 3D printing in prosthetics (N = 69, 34.5%), and CAD/CAM technology (computer-aided design/computer-aided manufacturing) (N = 84, 42.0%) are particularly notable.

Table 5 shows data on the use of specific innovations for practice management, preventive dentistry, patient behavior management, caries assessment, pain management, research, and data analyses. Electronic health records (EHRs) are utilized by the majority of respondents (N = 117, 58.5%) to manage their practice. Additionally, 12% of respondents (N = 24) use blockchain technology for patient printouts and consent.

**Figure 3:**
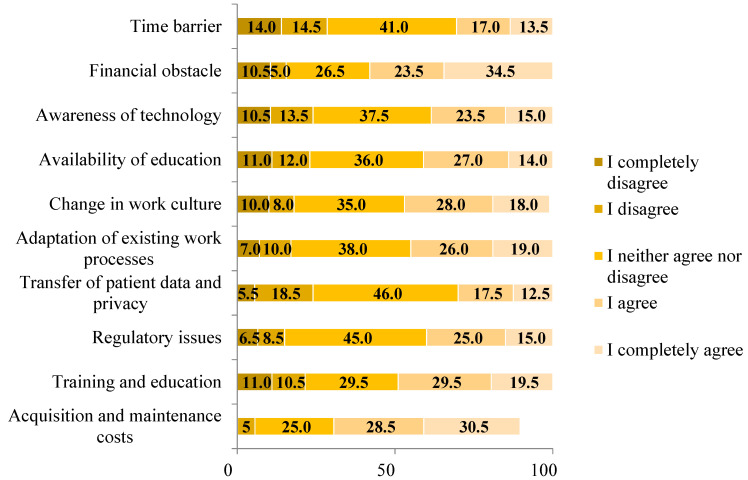
“What obstacle do you face in integrating modern technologies into your daily practice?” (% of responses).

Figure 3 displays the barriers encountered by participants when attempting to use modern technology and artificial intelligence in their clinical practice. The majority identified acquisition and maintenance costs (N = 119, 59.0%) and financial constraints (N = 116, 58.0%) as the primary obstacles. Additionally, 49.0% of participants (N = 98) viewed the availability of training and education as a barrier, while 25.0% (N = 59) remained neutral on this issue.

## 4. Discussion

Artificial intelligence can bring about revolutionary changes in the healthcare sector, including dentistry. It is used in diagnostics, treatment planning, and prognoses as well as risk assessment for certain diseases in dentistry. Although there is no doubt that the use of these technologies will continue to increase and evolve, there is a noticeable lack of knowledge throughout the field of dentistry [6,18].

The aim of this study was to assess the knowledge and use of modern technologies and artificial intelligence among dentists in the Republic of Croatia. A review of the literature on this topic revealed that no study has yet been conducted in this part of Europe. The number of participants who submitted their responses (N = 200) was in line with the minimum necessary sample size (N = 152). Based on the results obtained, it was found that the average knowledge of respondents about the application of artificial intelligence in dentistry is low, which can also be confirmed for the daily practice of using modern technologies and artificial intelligence in dental practices. Of the socio-demographic and professional characteristics examined, only the gender and educational level of the respondents had an influence on the level of knowledge examined.

Compared to men, women had a lower level of knowledge about the use of artificial intelligence in dentistry. In contrast to the results of this study, in which the practice of using modern technologies was at a low level, more than half of the respondents in a study in Saudi Arabia used digital technologies in their daily dental practice, and there were no significant differences in terms of gender [19]. A study in Poland also found no statistically significant differences in the use and knowledge of modern technologies between men and women and no differences by years of clinical experience [10]. Dentists with a PhD or a Master’s of Science degree showed greater knowledge than those who had only completed dental training, which could be explained by their more continuous training and long-term investment in knowledge. In another study in Saudi Arabia, students and interns demonstrated significantly greater knowledge than dentists with clinical experience. This is likely due to greater awareness of modern technologies through social media as well as student lectures, congresses, and continuing education, which respondents in this study cited as a way of acquiring knowledge about modern technologies and artificial intelligence [9].

Specialists were not included in this study, but it would be interesting to know their knowledge of these technologies, especially among specialists in dental prosthetics and oral surgery, as these are the areas where artificial intelligence and modern technologies are most used both in our study and according to Bernauer et al. [20]. The results of a study in the Netherlands show that doctors with a specialization have a greater knowledge and practice in the use of modern technologies, as well as those dentists who work more hours per week, in larger practices, and with more staff, and dentists in the younger age group [21].

In this study, no significant statistical differences in knowledge were found in relation to age, although it would be expected that younger people would have greater knowledge for the reasons already mentioned (better education in relation to technologies, better information through social media and other media). According to Gilakjani, age is an indicator that can influence individual use of new technologies [22]. Therefore, a study in Saudi Arabia examined the generational differences in the users of digital technologies in dentistry. Generation X integrated modern technologies into their clinical practice more effectively than Millennials (Generation Y). This finding suggests that Generation X dentists are likely to have gained more years of experience and exposure to digital technologies in dentistry compared to younger dentists [19].

Most respondents rated their current knowledge and understanding of modern technology and artificial intelligence as poor and moderate. In contrast to these data, a study conducted in India showed that their respondents evaluate their own knowledge about artificial intelligence and other modern technologies as good. Furthermore, respondents in the Indian study expressed a desire to increase their knowledge of these technologies [11]. This is in line with the results of our study, in which three quarters of respondents stated that they would like to participate in additional education and training in the future to better understand and use modern technologies and artificial intelligence more effectively. In a study in Kurnool, India, it was shown that most respondents believe that knowledge of the basic principles of artificial intelligence should be included in the curriculum or as an additional course during clinical practice [7]. A study carried out in the Netherlands confirms that dentists who make greater use of modern technologies in their clinical practice have a higher level of knowledge. The results of the same study also indicate that dentists who use modern technologies have a higher number of patients than colleagues who do not use them or use them to a lesser extent [21]. Despite findings from a 2024 systematic review indicating that dental students possess lower knowledge levels compared to practicing dentists (with the knowledge scale exceeding 70%), a larger proportion of these students believe that artificial intelligence will lead to significant advancements in the field of dentistry. While many dentists see the benefits of these technologies in the future, a smaller percentage believe that artificial intelligence can completely replace humans [23].

The respondents in this study use modern technology and artificial intelligence most frequently in prosthetics, oral surgery, and orthodontics. These findings are consistent with a systematic review of the use of artificial intelligence in dental prosthetics [20]. It has been shown that artificial intelligence is used for automated diagnostics and as a predictive measure with regard to treatment prognoses. In the broader field of prosthetics, artificial intelligence was most frequently used in CAD/CAM systems, implant prosthetics, and orofacial anatomy [20]. CAD/CAM systems were also the most common response in this study when it comes to the use of modern technology and artificial intelligence in patient therapy, along with intraoral scanners and 3D printing in prosthetics. Through the use of CAD/CAM technologies, various types of dental prostheses can not only be designed but also manufactured with exceptional precision and accuracy. Over the last 25 years, CAD/CAM technology has become extremely popular. The introduction and development of CAD/CAM technology in dentistry have revolutionized treatment methods and prosthetics. Although this technology is well established in fixed prosthetics, it is still a relatively new field in removable prosthetics [24]. It is believed that this technology has led to changes in the use of dental materials, such as the increased use of zirconia and lithium disilicate [12]. Similar results were obtained in a study in Romania, where most dentists responded that they use CAD/CAM technology in their clinical practice, namely more than half [25]. A study conducted in the UK shows that less than half of respondents use this technology in their clinical practice, suggesting that it is still relatively new and relatively underutilized in dentistry [12]. The use of new intraoral scanning systems has significantly reduced the time required for impression taking, and the accuracy and marginal fit of digital impression systems have recently been improved [26]. An intraoral scanner can increase patient cooperation by allowing the patient to follow the treatment in real time or via recordings, which is particularly useful for children and anxious patients [27]. The use of algorithms trained on databases that have shown similar effectiveness to experts would help oral surgery specialists to make treatment decisions (e.g., the extraction of impacted third molars, where these tools allow procedural guidance by predicting the complexity of the surgical procedure, detecting contact with the inferior alveolar canal on panoramic radiographs, and assessing the eruption potential of third molars by measuring their angulation). This would greatly facilitate the surgeon’s decision-making process [28]. When asked whether doctors knew in which areas of dentistry artificial intelligence and modern technologies could be used or are already being used, the most common answers were dental prosthetics for the design of restorations and orthodontics for treatment planning and predicting treatment outcomes. According to research in China, AI is most commonly used in orthodontics for diagnoses but has limited use in treatment. They believe that AI can assist orthodontists in correcting deep bites and preventing bone dehiscence or fenestration [29]. According to Borzabadi-Farahani et al., the use of AI to identify orthodontic treatment needs, such as the Index of Orthodontic Treatment Need (IOTN) and the Index of Functional Orthognathic Treatment Need (IOFTN), still requires further research [30].

According to Kalaimani et al., the results show that the respondents in their study believe that AI can be used in diagnoses, treatment planning, dental pathology (early detection of lesions, histopathologic analysis of cancer, etc.), prosthodontics (shorter treatment time, higher quality of work), and the interpretation of a cephalometric analysis [6]. These responses are consistent with this study, which asked similar specific questions about AI. They show that dentists believe that AI and other modern technologies can significantly support the treatment of their patients. However, they feel that they lack sufficient knowledge and skills in using these technologies. Therefore, awareness of AI should be raised by dental associations, research institutions, and technology companies by promoting discussions and educational resources on AI. Scientific papers, publications, and presentations on AI in dentistry can help raise awareness among dental students [6].

In this study, CBCT proves to be the most frequently used tool for diagnoses and treatment planning alongside digital imaging. The use of CBCT in dentistry is increasing exponentially. It allows precise measurements, better visibility of impacted tooth localization, the identification of endodontic problems, assessment of periodontal structures, and the verification of various oral anomalies. Despite its benefits, it is important to limit the use of CBCT to situations where the benefits outweigh the potential risks and to use it only when justified. This approach ensures better patient outcomes [31].

Most respondents in this study cited the costs of acquisition and maintenance, as well as financial barriers, as the main barriers to the use of AI and other advanced technology. Almost half of the respondents considered the availability of training and education to be a major barrier. This is in line with the results of a study conducted in the United Arab Emirates, where respondents also cited the availabilities of training and education as the biggest challenges and barriers to using these technologies [18]. In a study in Saudi Arabia, the biggest barriers to the introduction of digital technologies in clinical practice were the lack of awareness among practitioners, the lack of training, and the lack of initiators for the integration of modern technologies into clinical practice [19], while among dentists in Poland, high costs were the main reason for not using modern technologies and that is why Polish dentists prefer conventional treatment methods. The authors of the study believe that more research on this topic will help to better understand and comprehend the current state of modern technology in dentistry [10]. Lack of awareness is the main barrier to the use of AI in dentistry, according to a study by Nishi Sing et al. More than half of the participants stated that the lack of training at universities and the lack of technical resources are also obstacles to its use. They believe that better undergraduate training of professionals can help overcome future challenges in integrating modern technologies and AI [32].

This study had several limiting factors. The responses of the participants in this study may not represent the knowledge of all dentists in the Republic of Croatia, as the study was conducted at only one congress and with a relatively small number of participants. The study involved significantly more women than men, which has also been a problem in other studies on this topic, including the study by Hamd et al. in which almost twice as many women participated [18]. Furthermore, this study did not survey specialists, who may have more knowledge about modern technologies and AI because they are more focused on specific areas of expertise, may have more information, and may strive to improve the quality of their work. Furthermore, this is a cross-sectional study based on a questionnaire; that is, respondents provided a self-assessment of practice and knowledge, meaning their competences are defined and they can identify the gaps between their current knowledge level and desired level of knowledge by using rate scales.

To improve knowledge and increase the practice of using modern technology and AI, a better way to enrich our educational systems and encourage innovation and research is necessary. It is crucial to strive to improve the quality of care for our patients to make both patients and ourselves as satisfied as possible [33]. From studying the literature and other research on the same or similar topics, it can be concluded that every such study contributes to greater awareness and knowledge about innovations in modern technology. Therefore, more of such research should be conducted with an even larger number of participants. Also, considering that by reviewing similar studies, we found out that the amount of working hours affects the knowledge of artificial intelligence and modern technology, it would be good to add a question about it in the next studies.

It would be desirable for these studies to include a larger number of participants and be conducted at as many congresses, seminars, and events as possible, where a larger number of dental medicine doctors gather. Looking ahead, it will be essential to not only investigate the extent of AI utilization among dental professionals but also to explore additional factors that may influence their knowledge and acceptance of these technologies. This could involve analyzing demographic variables, educational backgrounds, and previous experiences with technology in clinical settings.

## 5. Conclusions

The study reveals, in line with other research across various countries, that most respondents have limited knowledge of artificial intelligence in dentistry, with women and dentists without postgraduate education showing a lower level of understanding. Although the majorities do not currently use these technologies in clinical practice, they recognize their potential to improve patient care. High costs and limited access to training are major barriers. To address this issue, dental institutions and organizations should provide more accessible, affordable AI training, including online courses and hands-on workshops. In addition, financial incentives or subsidies for adopting new technologies could encourage wider use of AI, helping to close the knowledge gap and improve clinical outcomes.

## Figures and Tables

**Table 1:** Socio-demographic and professional characteristics.

Characteristics	Choice	Total N (%)	AI—Knowledge	*p*-Value
Sex	Male	52 (25.5)	Reference	
	Female	149 (74.5)	−1.170 (−1.883–−0.457)	≤0.001
Age group (years)	23–40	120 (60.0)	Reference	
	41–55	60 (30.0)	−0.563 (−2.196–1.070)	0.499
	>56	20 (10.0)	−0.363 (−1.592–0.856)	0.556
Working experience (years)	1–10	104 (52.0)	Reference	
	11–20	47 (23.5)	0.345 (−0.713–1.403)	0.523
	>21	49 (24.5)	−0.254 (−1.720–1.212)	0.734
Education level	DMD	179 (89.5)	Reference	
	MSc	13 (6.5)	−0.431 (−2.018–1.156)	0.595
	PhD	8 (4.0)	1.676 (0.219–3.134)	0.024
Type of healthcare practice	Community	94 (47.0)	Reference	
	Concession (semiprivate)	28 (14.0)	0.759 (−0.193–1.172)	0.118
	Private	78 (39.0)	0.136 (−0.557–0.830)	0.700

Data are presented as numbers (percentages, %). Reference knowledge category is “low”. β, regression coefficients; 95% CI, 95% confidence interval (*p* ≤ 0.05). Abbreviation: AI—artificial intelligence.

**Table 2:** Self-assessment of the use of modern technology and AI in clinical practice.

Question		Total N (%)	AI—Knowledge	*p*-Value
Self-assessment of personal knowledge about modern technologies and AI in dentistry	Very poor	16 (8.0)	Reference	
	Poor	56 (28.0)	−0.065 (−1.598–1.468)	0.934
	Average	73 (36.5)	0.565 (−0.963–2.094)	0.469
	Good	46 (23.0)	0.632 (−1.052–2.317)	0.462
	Excellent	9 (4.5)	0.216 (−2.790–3.042)	0.932
Perceived education in AI and modern technology topics during medical graduate and postgraduate studies	Yes	121 (60.5)	0.178 (−0.649–1.006)	0.673
	No	79 (39.5)	Reference	
Availability and affordability of training and education on modern technologies in dental practice	Very poor	7 (3.5)	Reference	
	Poor	60 (30.0)	−1.359 (−3.852–1.062)	0.266
	Average	82 (41.0)	−1.359 (−3.788–1.070)	0.273
	Good	44 (22.0)	−2.093 (−4.629–0.444)	0.106
	Excellent	7 (3.5)	20.251 (−54,309.759–4350.261)	0.999
Treatment quality and patient experience can be enhanced through the integration of AI systems and modern technology into dental practice	Very poor	4 (2.0)	Reference	
	Poor	31 (15.5)	−0.782 (−3.643–2.079)	0.592
	Average	72 (36.0)	−1.513 (−4.254–1.228)	0.279
	Good	69 (34.5)	−1.579 (−4.385–1.226)	0.270
	Excellent	24 (12.0)	−0.747 (−3.736–2.243)	0.624
Potential of using clinical AI systems in clinical practice for supporting decision making	Yes	108 (54.0)	0.072 (−1.284–1.427)	0.917
	No	27 (13.5)	Reference	
	I don’t know	65 (32.5)	−1.245 (−2.621–0.131)	0.076
Interested in further education on topic of AI and modern technology	Yes	157 (78.5)	2.607 (0.259–4.956)	0.030
	No	13 (6.5)	Reference	
	I don’t know	30 (15.0)	1.733 (−0.639–4.105)	0.152
Believe that modern technology and AI can improve the quality of treatment	Yes	142 (71.0)	−0.203 (−2.247–1.841)	0.845
	No	11 (5.5)	Reference	
	I don’t know	47 (23.5)	−1.416 (−3.498–0.665)	0.182

Data are presented as numbers (percentages, %). Reference knowledge category is “low”. β, regression coefficients; 95% CI, 95% confidence interval (*p* ≤ 0.05). Abbreviation: AI—artificial intelligence.

**Table 3:** Practice of using modern technology in diagnostics and treatment planning.

Category	Application	Yes	No
Diagnosis and treatment planning	Digital imaging	142 (71.0)	58 (29.0)
	Cone beam computed tomography (CBCT) systems	134 (66.0)	67 (33.0)
	AI analysis of X-ray images (artificial intelligence)	32 (16.0)	168 (84.0)
	Software for gathering insights into clinical decision making (AI)	17 (8.5)	183 (91.5)
	Intraoral camera	71 (35.5)	129 (64.5)
	3D scanning—orthodontics	48 (24.0)	152 (76.0)
	3D scanning—oral surgery	47 (23.5)	153 (76.5)
	3D scanning—mobile prosthodontics	45 (22.5)	155 (77.5)
	3D scanning—fixed prosthodontics	70 (35.0)	130 (65.0)
	AI-guided treatment planning in orthodontics	26 (13.0)	174 (87.0)
	Operating microscope	16 (8.0)	184 (92.0)
	Digital pathology	11 (5.5)	189 (94.5)
	Modern devices for oral cancer screening (ViziLite^®^, VELscope^®^, OralCDx^®^ BrushTest^®^, Identafi^®^, fluorescence system, Biospec^®^)	11 (5.5)	189 (94.5)

Data are presented as numbers (percentages, %). Abbreviation: AI—artificial intelligence.

**Table 4:** Practice of using modern technology in therapy.

Category	Application	Yes	No
Therapy	Computer-assisted surgery (CAS)	21 (10.5)	179 (89.5)
	Virtual surgical planning (VSP)	26 (13.0)	174 (87.0)
	Robot-assisted surgery	7 (3.5)	193 (96.5)
	3D-guided implant surgery	34 (17.0)	166 (83.0)
	Intraoral scanners for implantology	48 (24.0)	152 (76.0)
	Intraoral scanners for mobile prosthodontics	43 (21.5)	157 (78.5)
	Intraoral scanners for fixed prosthodontics	63 (31.5)	137 (68.5)
	Intraoral scanners for orthodontics	43 (21.5)	157 (78.5)
	3D printing in implantology	43 (21.5)	157 (78.5)
	3D printing in prosthetics	69 (34.5)	131 (65.5)
	3D printing in orthodontics—customized braces	20 (10.0)	180 (90.0)
	Electromyography (EMG)	7 (3.5)	193 (96.5)
	Bioprinting	10 (5.0)	190 (95.0)
	Microosteoperforations—minimally invasive procedures that speed up tooth movement	10 (5.0)	190 (95.0)
	Digital Smile Design	52 (26.0)	148 (74.0)
	CAD/CAM (computer-aided design/computer-aided manufacturing) technology	84 (42.0)	116 (58.0)
	Virtual articulator	27 (13.5)	173 (86.5)
	Ozone	23 (11.5)	177 (88.5)
	Laser	37 (18.5)	163 (81.5)

Data are presented as numbers (percentages, %).

**Table 5:** Practice of using modern technologies in dentistry.

Category	Application	Yes	No
Office management	Electronic health records (EHRs)	117 (58.5)	83 (41.5)
	Scheduling appointments using artificial intelligence	21 (10.5)	179 (89.5)
Preventive dental medicine	AI—Enhanced Monitoring—oral hygiene habits	14 (7.0)	186 (93.0)
Patient behavior management	Virtual Reality (VR) and Augmented Reality (AR)	16 (8.0)	184 (92.0)
	Sedation—nitrous oxide—gas	16 (8.0)	184 (92.0)
	Sedation—oral conscious sedation—pill	22 (11.0)	178 (89.0)
	Sedation—NuCalm technique—neuromuscular technique	9 (4.5)	191 (85.5)
Caries assessment	Laser systems based on fluorescence/DIAGNOdent	19 (9.5)	181 (90.5)
	Digital fiber optic translumination (DIFOTI)	17 (8.5)	183 (91.5)
	AI algorithms	8 (4.0)	192 (96.0)
Patient discharges and consent	Blockchain	24 (12.0)	176 (88.0)
Pain management	Computer-controlled anesthesia	9 (4.5)	191 (95.5)
Research and data analysis	AI in dental research	12 (6.0)	188 (94.0)
	Big Data analysis	12 (6.0)	188 (94.0)

Data are presented as numbers (percentages, %). Abbreviation: AI—artificial intelligence.

## Data Availability

The data that support the findings of this study are available upon request from the corresponding author.
